# Differences and characteristics in amino acids, catechins, and flavonoids among alfalfa (*Medicago sativa* L.) germplasms and tissues according to UHPLC-Q Exactive-MS technology

**DOI:** 10.3389/fpls.2026.1791873

**Published:** 2026-04-23

**Authors:** Tianhui Yang, Liyue Yang, Ping Liu, Xueqian Jiang, Fei He, Chuan Wang, Junmei Kang, Qingchuan Yang, Xizhe Zhu, Ping Li, Ting Gao, Jinjie Hua

**Affiliations:** 1 Institute of Grassland Research, Chinese Academy of Agricultural Sciences, Key Laboratory of Forage Resources and Utilization, Ministry of Agriculture and Rural Affairs, Hohhot, Inner Mongolia, China; 2 Institute of Animal Science, Ningxia Academy of Agricultural and Forestry Sciences, Yinchuan, Ningxia, China; 3 Tea Research Institute, Chinese Academy of Agricultural Sciences, State Key Laboratory of Tea Plant Germplasm Innovation and Resource Utilization, Hangzhou, Zhejiang, China; 4 Hangzhou Jingshan Tea Industry Co., Ltd, Hangzhou, Zhejiang, China; 5 Institute of Animal Science, Chinese Academy of Agricultural Sciences, Beijing, China

**Keywords:** alfalfa (*Medicago sativa* L.), amino acids, catechins, flavonoids, germplasms and tissues

## Abstract

Alfalfa (*Medicago sativa* L.) is one of the most widely planted forages worldwide. It is rich in anthocyanins, polyphenols, amino acids, and other functional flavor non-volatile metabolites (NVMs). Its various germplasms and plant parts differ markedly with regard to quality components; however, a rigorous assessment of these differences is lacking, which hampers the optimization and diversified use of alfalfa. Using a targeted metabolomics approach based on UHPLC-Q Exactive-MS, we absolutely quantified 60 non-volatile metabolites (including catechins, flavonoids, amino acids, alkaloids, theaflavins, and phenolic acids) in the stem, leaf, and flower tissues of eight alfalfa germplasms, among which 43 were identified as alfalfa-associated quality NVMs. Six catechin components (epigallocatechin gallate [EGCG], catechin [C], epicatechin [EC], epigallocatechin [EGC], gallocatechin gallate [GCG], and epicatechin gallate [ECG]) were first identified, of which EGCG showed the highest content (0.241 mg/g). Overall, stem tissue was rich in catechins and phenolic acids, flower tissue was rich in flavonoids, SAAs, BAAs, and OAAs, and leaf tissue was rich in UAAs and (UAAs+SAAs)/BAAs. Meanwhile, 35zg germplasm stem was rich in catechins, 207kc flower was rich in flavonoids, phenolic acids, and BAAs, 260dy flower was rich in SAAs and OAAs, and 207kc leaf was rich in UAAs. A total of 18 differential NVMs, including 10 amino acids, 4 catechins, and 4 flavonoids, were screened according to the significant difference values, variable importance in projection, heatmapping, and classification analyses. Tissue classification analysis showed that alfalfa stems and leaves were rich in glutamic acid (producing an umami taste), EGCG, catechin (C), epicatechin (EC), and epigallocatechin (EGC) (with an intense taste). Meanwhile, flowers were rich in prunin, myricetin, and quercetin 3-O-D-glucose-7-O-D-gentiobioside (producing an astringent taste), whereas valine, leucine, isoleucine, methionine, citrulline, and histidine resulted in a bitter taste. Germplasm classification analysis showed that 35zg was confirmed to be rich in EGCG, C, EC, and EGC (with an intense taste), and the L-tyrosine, valine, leucine, and citrulline contents (which result in a bitter and astringent taste) were low. Our results provide theoretical and technical support for the characteristic analysis and diversified utilization of alfalfa materials and the development of characteristic herbal tea products.

## 1 Introduction

Alfalfa (*Medicago sativa* L.) is one of the most widely cultivated legumes globally predominantly for forage, but is also used for herbal tea, owing to its richness in health-beneficial compounds, such as flavonoids, polyphenols, amino acids, minerals (including iron, phosphorus, potassium, and magnesium), and vitamins (including vitamins A, D, E, G, and K) (Ames et al., 1995; Bhattarai et al., 2021; Chen et al., 2020). Furthermore, alfalfa can exert various beneficial effects on health, such as eliminating arthritis, helping digestion, treating gastric ulcers, and relieving cough and alcohol (Baragaño et al., 2022; Chen et al., 2024; Cunningham and Volenec, 1996). Alfalfa is widely planted all over the world. Its mainly produced in areas with suitable climate and developed animal husbandry, such as the United States and Canada in North America; Spain, Italy, France, and Greece in Europe; China and Kazakhstan in the Asia Pacific region; Argentina and Chile in South America. North America, Europe, and the Asia Pacific region together account for more than 85% of the global consumption share. By 2025, the planting area of Alfalfa in China had reached about 2 million ha, with the annual output of alfalfa hay in China exceeding 10 million tons. The overall growth of the global alfalfa market is relatively stable, and China is one of the fastest-growing markets for alfalfa. Although China is a large producer, there is still a structural shortage of high-quality alfalfa (particularly high-grade grass with protein content >18%). The alfalfa industry occupies a core position in China’s grass and animal husbandry industry, serving as the key foundation to support the high-quality development of the dairy industry. Furthermore, it is rich in dietary fiber (Feng et al., 2022; Guo et al., 2024; Wu et al., 2021), low in calories, and a good food and drink with high fiber and low calories, which gives it a wide prospect of applications in the field of health care and consumer markets.

Long-term artificial breeding and natural adaptation have resulted in pronounced diversity among alfalfa germplasms in terms of genetics and metabolomics, including polyphenol and flavonoid content and composition (Bakhtiar et al., 2025; Lu et al., 2025; Wei et al., 2025; Zi et al., 2025). For example, some germplasm with strong cold resistance or disease resistance, such as *Grassland* series, tend to accumulate higher levels of quercetin, kaempferol, and their glycoside derivatives with antioxidant activity (Chen et al., 2024; Ferchichi et al., 2023; Wu et al., 2023). These compounds are the chemical bases of stress resistance physiological function, and produce a bitter and astringent taste. In addition, amino acids are important antinutritional factors and bioactive non-volatile metabolites (NVMs) in alfalfa (Fiutak et al., 2019; Guo et al., 2024; He et al., 2025), and their components and contents vary markedly among different germplasms, which directly affects palatability and potential health benefits.

Metabolite synthesis, accumulation, and storage are highly tissue-specific processes that differ among functionally differentiated organs, such as flowers, leaves, and stems. The leaf is the primary site of photosynthesis and secondary metabolism in alfalfa. Thus, the leaves are typically rich in NVMs, including proteins, vitamins, chlorophylls, and flavonoids (He et al., 2025; Ma et al., 2025; Shang et al., 2021); these NVMs produce the fresh and slightly astringent “grassy” taste of alfalfa and constitute its main antioxidant and anti-inflammatory functions. The main function of the stem is physical support and transportation, thus the content of structural carbohydrates, such as cellulose and hemicellulose, is high (Li et al., 2023; Müller et al., 2025; Veljković et al., 2024; You et al., 2022), whereas the content of proteins and active secondary metabolites is relatively low. The stem is thus the main source of dietary fiber, but has little flavor. The flowers are reproductive organs with active metabolic activities, and are typically rich in anthocyanins, flavonoids, and precursors of volatile metabolites (VMs) (Liu et al., 2018; Smirnova et al., 2022; Wang Q. D. et al., 2021), as well as some unique amino acids; these NVMs produce a richer flavor and sweet taste.

Studies on alfalfa have mostly focused on agronomic shape, genome analysis, functional gene mining, germplasm development, and physiological stress. For example, through the analysis of 291 alfalfa agronomic traits and genes under salt stress, 49 SNP loci significantly associated with salt tolerance traits were identified, and 21 candidate genes associated with salt tolerance traits were screened out (Bhattarai et al., 2021; Guo et al., 2024). Through functional gene mining, the dehydrated protein CsLEA was screened from alfalfa, and showed increased chlorophyll content and leaf relative water content, reduced Na^+^ content, and enhanced drought and salt tolerance (Ferchichi et al., 2023; Ma et al., 2025; Zhang et al., 2023). Based on the phenotypic association analysis of 45 agronomic traits of 137 alfalfa materials from the U.S. Germplasm Bank, 33 SNP molecular markers associated with salt tolerance traits were screened out (Shen et al., 2020); some of the important traits include salt tolerance, insect resistance, yield, and disease resistance. However, few studies have addressed the analysis and mining of functional flavor NVMs and VMs in alfalfa germplasms and none have examined the differences in these compounds among tissues, which hamper the optimization, diversified use, and industrial application prospects of alfalfa germplasms.

In the present study, different tissues (stems, leaves, and flowers) of eight representative alfalfa germplasms were examined. The quality of NVMs components, such as catechins, flavonoid glycosides, amino acids, alkaloids, and phenolic acids, was quantitatively determined using ultra-high performance liquid chromatography (UPLC) with Q Exactive mass spectrometry (MS), and the variations in flavor NVMs in different tissues of different germplasms were analyzed based on significant difference values, variable importance in projection (VIP), heatmapping, and classification analyses. Key biochemical indicators that characterize the flavor differences of different alfalfa germplasms and tissues were screened, and their distribution in the various germplasms and tissues was explored to screen useful alfalfa germplasms and tissues that could be used for new alfalfa herbal tea products. This research will provide theoretical support for the high-quality and efficient utilization of alfalfa germplasms, while offering technical support for breeding high-quality alfalfa varieties.

## 2 Materials and methods

### 2.1 Sample preparation

**Figure 1:**
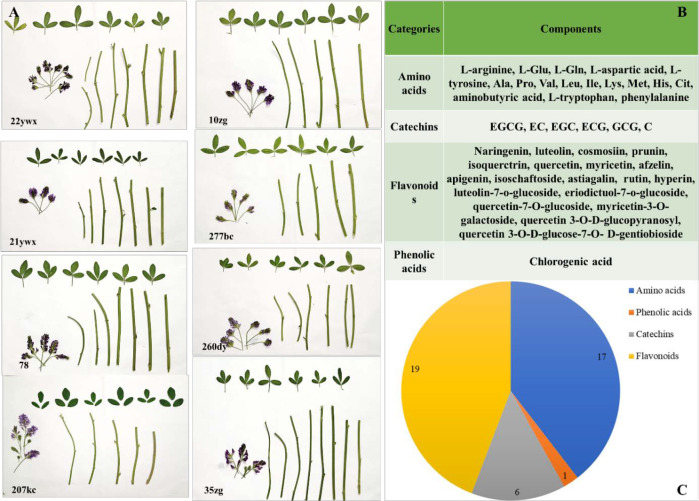
Basic information of flower, stems and leaves appearance and the distribution of non-volatile metabolites (NVMs) in eight alfalfa (*Medicago sativa L.*) germplasms. **(A)** appearance photos, **(B)** NVMs categories and components, **(C)** NVMs categories numbers.

The flowers, stems, and leaves of eight alfalfa germplasms (i.e., 22ywx, 10zg, 21ywx, 277bc, 150hj, 260dy, 35zg, and 207kc) were harvested in May 2024 (**Figure 1A**).

The Flowers were in the budding stage. Leaves were harvested during the fresh tender stage, and stems between the first and second leaves were harvested in the tender stem stage. All raw materials were collected at the research station of the Ningxia Academy of Agricultural and Forestry Sciences (38.21° N, 106.22° E), Yinchuan, Ningxia province, China. The samples were transported in with dry ice and then freeze-dried, ground, sieved, and stored at -20 °C until testing.

### 2.2 Quantitative detection of quality NVMs

The quality NVMs were quantitatively detected using targeted metabolomics with UHPLC-Q Exactive-MS according to a previously published method (Wang H. J. et al., 2021; Yu et al., 2024). Alfalfa samples (0.2 g) were weighed and added to 10 mL 70% methanol for 10 min. After centrifugation at 4 °C and 10,000 r/min for 10 min, the supernatant was filtered through a 0.22 µm filter membrane and placed in a liquid-phase vial for testing. A Waters ACQUITY UPLC H-class system (Milford, MA, USA) coupled to a Waters Xevo TQ-S Micro triple-quadrupole mass spectrometer was used for the UHPLC-Q Exactive-MS analysis. A Waters ACQUITY UPLC HSS T3 analytical column (1.8 µm, 2.1 mm × 100 mm) was used at a temperature of 40 °C. The sample injection volume was 5 µL. The mobile phase consisted of 0.1% (v/v) formic acid in water (A) and 0.1% (v/v) formic acid in acetonitrile (B). The mobile phase gradient was set as follows: 0 min, 95% A and 5% B at a flow rate of 0.25 mL/min; 0.5 min, 85% A and 15% B at a flow rate of 0.25 mL/min; 5 min, 75% A and 25% B at a flow rate of 0.25 mL/min; 9 min, 35% A and 65% at a flow rate of 0.25 mL/min; 9.8 min, 0% A and 100% B at a flow rate of 0.25 mL/min; 10.8 min, 0% A and 100% at a flow rate of 0.40 mL/min; and 11 min, 95% A and 5% B at a flow rate of 0.40 mL/min. MS/MS analysis was conducted using electrospray ionization in the positive ion mode. The mass-to-charge ratio and retention times of flavonoid, catechin, amino acid, alkaloid, and phenolic acid components are shown in **Supplementary Table 1**. The contents of 60 quality NVMs (8 catechins, 19 amino acids, 2 alkaloids, 4 theaflavins, 1 phenolic acid, and 26 flavonoids) were absolutely quantified using the calibration curves of their corresponding standard solutions, and 43 NVMs were associated with alfalfa.

### 2.3 Statistical analysis

The tests were conducted in three replicates, and the results are expressed as the means of the three replicates. Origin software (version 8.0; Origin Lab Co., MA, USA) was used to create plots visualizing NVMs differences among germplasms and tissues. SPSS (version 22.0; IBM, Armonk, NY, USA) was used for statistical analyses among samples. SIMCA 14.1 (Umetrics, Umea, Sweden) was used for partial least squares discriminant analysis (PLS-DA), and mining differential NVMs among samples. The Matware data platform (https://cloud.metware.cn) was used to create a heat map of the differential NVMs.

## 3 Results and discussion

### 3.1 Overall analysis of NVMs among alfalfa (*Medicago sativa* L.) germplasms and tissues

#### 3.1.1 Overall analysis

Based on UHPLC-Q Exactive-MS technology, 43 NVMs were quantitatively detected and identified in three tissue parts of eight alfalfa germplasms (**Figures 1B, C**), including 19 flavonoids and flavonoid glycosides (naringenin, luteolin, cosmosiin, prunin, isoquerctrin, quercetin, myricetin, afzelin, apigenin, isoschaftoside, astiagalin, rutin, hyperin, luteolin-7-o-glucoside, eriodictuol-7-o-glucoside, quercetin-7-O-glucoside, myricetin-3-O-galactoside, quercetin 3-O-D-glucopyranosyl, and quercetin 3-O-D-glucose-7-O-D-gentiobioside); 17 amino acids (L-arginine, glutamic acid [L-Glu], glutamine [L-Gln], L-aspartic acid, L-tyrosine, alanine [Ala], proline [Pro], valine [Val], isoleucine [Ile], leucine [Leu], lysine [Lys], histidine [His], citrulline [Cit], aminobutyric acid, L-tryptophan, and phenylalanine); 6 catechins (epigallocatechin gallate [EGCG], catechin [C], epicatechin [EC], epigallocatechin [EGC], gallocatechin gallate [GCG], and epicatechin gallate [ECG]); and one phenolic acid (chlorogenic acid).

#### 3.1.2 Comparative analysis of different NVMs types

**Figure 2:**
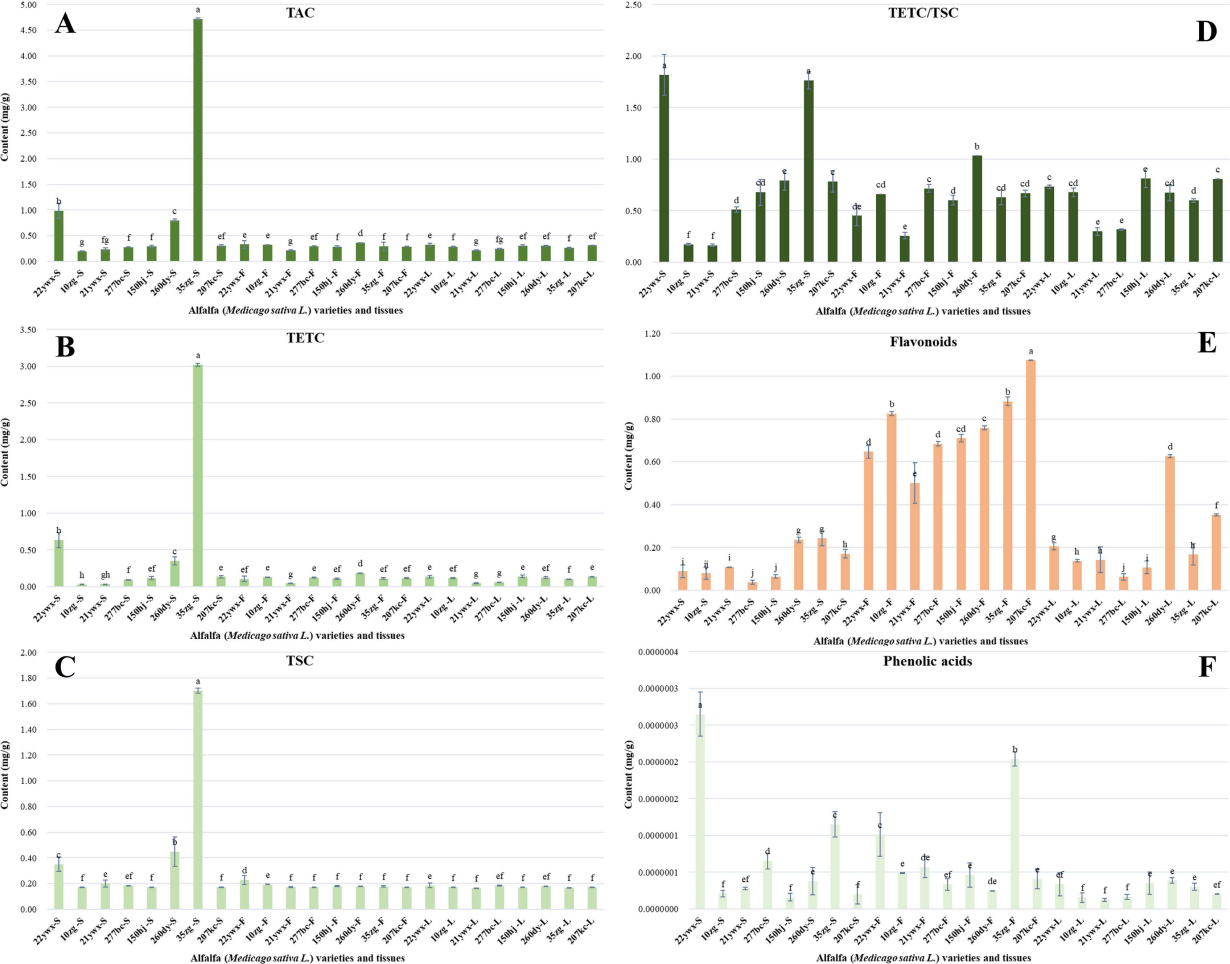
Overall analysis of non-volatile metabolites (NVMs) among alfalfa (*Medicago sativa L.*) germplasms and tissues. **(A)** total amount of catechins (TAC) content, **(B)** total ester type catechins (TETC) content, **(C)** total simple catechins (TSC) content, **(D)** TETC/TSC value, **(E)** total amount of flavonoids content, **(F)** total amount of phenolic acids content.

**Figure 3:**
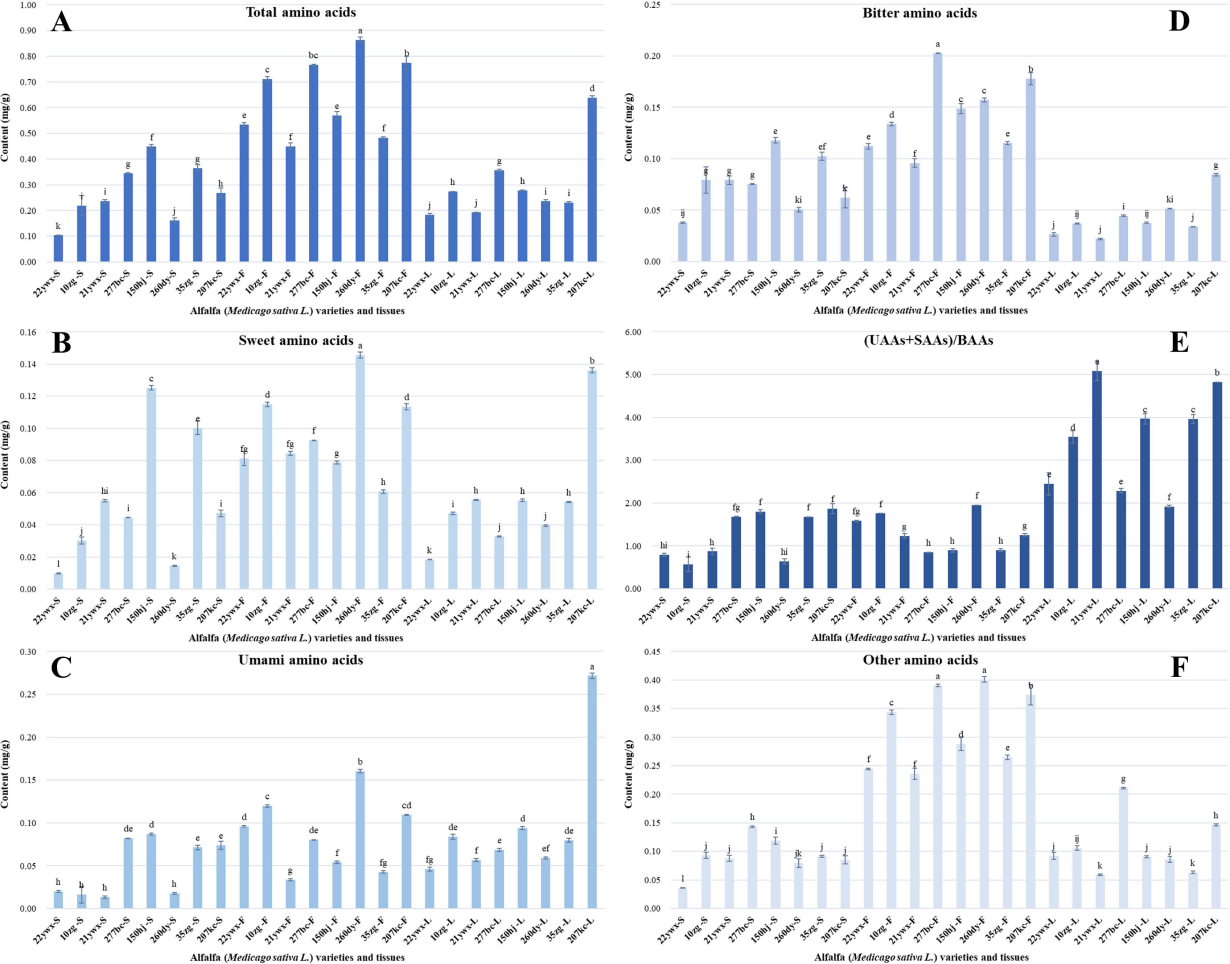
Analysis of amino acids types among alfalfa (*Medicago sativa L.*) germplasms and tissues. **(A)** total amount of amino acids content, **(B)** sweet amino acids (SAAs) content, **(C)** umami amino acids (UAAs) content, **(D)** bitter amino acids (BAAs) content, **(E)** (UAAs + SAAs)/BAAs value, **(F)** other amino acids content.

The content of NVMs with various flavor characteristics differed significantly between plant parts and germplasms (**Figures 2**, **3**; **Supplementary Table 1**). Catechins are phenolic active substances with antitumor, antioxidant, disease-resistant, and other health benefits (Hua et al., 2021; Wang et al., 2022a). These key NVMs determine tea taste quality and impart strong astringency. Catechins are classified as simple catechins (TSC) and ester-type catechins (TETC) (Yang et al., 2025; Zhu et al., 2025).

TETC has a strong bitter and astringent taste, whereas TSC has a weak bitter and astringent taste. We identified six catechins in alfalfa germplasms for the first time (Ferchichi et al., 2023; Feng et al., 2022). Among the different catechin components (**Supplementary Table 1**), the EGCG content was the highest, with an average of 0.241 mg/g, followed by EGC, with an average of 0.177 mg/g. C was the third highest, with an average of 0.048 mg/g, and the content of GCG was the lowest. The amount of total catechins (TAC) was the highest in the stem (**Figure 2A**), with an average of 0.977 mg/g; there was no significant difference between the flower and leaf tissues. TAC content differed significantly among germplasms: it was the highest in 35zg stem (4.719 mg/g), followed by 22ywx stem (0.984 mg/g) and 260dy stem (0.800 mg/g). The contents in other germplasms and tissues were all below 0.370 mg/g, and that in 10zg stem tissue was the lowest (0.201 mg/g).

Among different tissues, TETC content was the highest in stem tissue (**Figure 2B**; mean, 0.551 mg/g); TETC content did not differ significantly between the flower and leaf tissues, at approximately 0.100 mg/g. The difference in TETC among different germplasms was more pronounced than that of TAC, ranging from 0.020 mg/g to 3.017 mg/g, and it was the highest in 35zg stem (3.017 mg/g), followed by 22ywx stem (0.634 mg/g) and 260dy stem (0.350 mg/g). The contents in other germplasms and tissues were all below 0.185 mg/g, without significant differences. It was the lowest in 21ywx, and the contents in stems, flowers, and leaves were all below 0.050 mg/g.

TSC content was the highest in stem tissue (**Figure 2C**; mean, 0.425 mg/g); there was no significant difference between the flowers and leaves, with a content of approximately 0.180 mg/g. The difference in TSC among different germplasms was less pronounced than that of TAC, ranging from 0.166 mg/g to 1.702 mg/g; it was the highest in 35zg stem tissue (1.702 mg/g), followed by 260dy stem tissue (0.450 mg/g), and 22ywx stem tissue (0.350 mg/g), 22ywx flower tissue (0.228 mg/g); the contents in other germplasms and tissues were between 0.166 mg/g and 0.200 mg/g, with no significant difference. The leaf tissue of the 21ywx germplasm was the lowest, at only 0.166 mg/g.

The TETC/TSC ratio (**Figure 2D**) is an important index for evaluating tea taste quality (Wang et al., 2024; Yu et al., 2023). The higher the ratio, the more bitter and astringent the taste. Among the different tissues, TETC/TSC was the highest in stem tissue (**Figure 2D**), with an average of 0.936. There was no significant difference between flower and leaf tissues, at approximately 0.610. Among germplasms, TETC/TSC ranged from 0.164 to 1.818; it was the highest in 22ywx stem tissue (1.818), followed by 35zg stem tissue (1.763) and 260dy flower tissue (1.032). The ratios in other germplasms and tissues were all below 1.000, and there were significant differences; it was the lowest in 21ywx stem tissue (0.164).

Flavonoids have a very low taste threshold, and are widely distributed across all plant types. Flavonoids are important health-functional NVMs, and also produce astringency (Ouyang et al., 2024; Wang H. J. et al., 2021). We identified 19 flavonoid components in the alfalfa germplasm. Among them (**Supplementary Table 1**), the isoschaftoside content was the highest (mean, 0.121 mg/g), followed by quercetin 3-O-D-glucopyranosyl (mean, 0.055 mg/g), astiagalin (mean, 0.044 mg/g), and rutin (mean, 0.0002 mg/g). Their content in flower tissues was the highest (**Figure 2E**), with an average of 0.761 mg/g; their content in all germplasm flower tissues exceeded 0.500 mg/g, followed by leaf tissues (mean, 0.225 mg/g) and stem tissues (mean, 0.129 mg/g). There were significant differences among the different germplasms, with flavonoid content ranging from 0.038 mg/g to 1.075 mg/g; it was the highest in 207kc flower tissue (1.075 mg/g), followed by 35zg flower tissue (0.883 mg/g), 10zg flower tissue (0.825 mg/g), and 277bc stem tissue (0.038 mg/g).

Phenolic acids are important NVMs that produce a sour taste (Hua et al., 2024a; Ouyang et al., 2024). Their levels were significantly higher in stem and flower than in leaf tissue (**Figure 2F**). Differences among germplasms were more pronounced in stem and flower tissues than in leaf tissues. Phenolic acid content was the highest in 22ywx stem tissue, followed by 35zg flower tissue; it was the lowest in 21ywx leaf tissue.

Amino acids are the key NVMs that determine plant taste and can be divided into umami amino acids (UAAs), sweet amino acids (SAAs), bitter amino acids (BAAs), and other amino acids (OAAs), according to their taste characteristics (Smirnova et al., 2022; Wang et al., 2022a, 2024). Seventeen amino acid components were identified in the alfalfa germplasms (**Supplementary Table 1**). L-tryptophan content was the highest (mean, 0.150 mg/g), followed by L-Glu (mean, 0.054 mg/g) and Pro (mean, 0.047 mg/g); that of Lys was the lowest (mean, 0.0001 mg/g). Flower tissue had the highest amino acid content (**Figure 3A**; mean, 0.644 mg/g), and it exceeded 0.450 mg/g in the flower tissue of each germplasm, followed by leaf tissue (mean, 0.299 mg/g), and it was the lowest in stem tissue (mean, 0.268 mg/g). The amino acid content differed markedly among the various germplasms, ranging from 0.103 mg/g to 0.864 mg/g. It was the highest in 260dy flower tissue (0.864 mg/g), followed by 207kc leaf tissue (0.775 mg/g), 277bc flower tissue (0.766 mg/g); it was significantly the lowest in 22ywx stem (0.103 mg/g).

SAAs include Ala, Pro, and Met (Yang et al., 2025). Overall, SAAs content ranged from 0.010 mg/g to 0.146 mg/g, accounting for approximately 17.0% of the total amino acids. Among tissues, the flower tissue had the highest SAAs content (**Figure 3**; mean, 0.096 mg/g), and the SAAs content in all germplasm flower tissues exceeded 0.061 mg/g. There was no significant difference between leaf and stem tissues, with an average content of approximately 0.054 mg/g. The difference among different tissues of different germplasms was more than 10-fold, and the content in 260dy flower tissue was the highest (0.146 mg/g), followed by 207kc leaf tissue (0.136 mg/g) and 150hj stem tissue (0.125 mg/g); it was the lowest in 22ywx stem tissue (0.010 mg/g).

UAAs include L-Glu, L-Gln, and L-aspartic acid (Zhu et al., 2025). Overall, the UAA content ranged from 0.013 mg/g to 0.272 mg/g, accounting for approximately 19.0% of the total amino acids. With regard to tissues, the highest UAAs content occurred in leaves (**Figure 3C**; mean, 0.095 mg/g), followed by flowers (mean, 0.087 mg/g) and stems (mean, 0.048 mg/g). The UAAs content differed markedly among tissues of various germplasms, with a difference of more than 20-fold. The content in 207kc leaf tissue was the highest (0.272 mg/g), followed by 260dy flower tissue (0.160 mg/g) and 10zg flower tissue (0.120 mg/g); it was the lowest in 22ywx stem tissue (0.013 mg/g).

BAAs include L-arginine, L-tyrosine, aminobutyric acid, Val, Leu, Ile, Lys, and His (Wang et al., 2024). Overall, BAAs content ranged from 0.022 mg/g to 0.203 mg/g, accounting for approximately 21.0% of total amino acids. The highest content occurred in flower tissue (**Figure 3D**; mean, 0.143 mg/g), followed by stem tissue (mean, 0.076 mg/g), and the lowest BAAs content was in leaf tissue (mean, 0.042 mg/g). BAAs content differed markedly among tissues of the various germplasms, and the difference was more than 10-fold. It was the highest in 277bc flower tissue (0.203 mg/g), followed by 207kc flower tissue (0.178 mg/g), and 260dy flower tissue (0.157 mg/g). The BAAs content of more than half of the alfalfa germplasms was less than 0.100 mg/g, and that of 21ywx leaf tissue was the lowest (0.022 mg/g).

To comprehensively evaluate the contribution of amino acids in different tissues of different germplasm lines, the (UAAs+SAAs)/BAAs value was introduced (Yang et al., 2025; Yu et al., 2024; Zhu et al., 2025). (UAAs+SAAs)/BAAs differed significantly among tissues of the various germplasms, ranging from 0.571 to 5.085. The ratio in leaf tissue (**Figure 3E**; mean, 3.505) was significantly higher than that in flower and stem tissue, and the ratio in all leaf tissues was more than 2.0, whereas that in stem and flower tissues was less than 2.0. The (UAAs+SAAs)/BAAs value showed significant differences among different tissues of different germplasms. The ratio was the highest in 21ywx leaf tissue (5.085), followed by 207kc leaf tissue (4.825) and 150hj leaf tissue (3.967). The (UAAs+SAAs)/BAAs value of more than half of the alfalfa germplasms was more than 1.200, and the lowest occurred in 10zg leaf tissue (0.570). Overall, the umami and sweet taste of amino acids in alfalfa leaf tissue were significantly higher than the bitter taste, indicating that alfalfa leaves had the potential to be used in drinking products.

OAAs include L-tryptophan, phenylalanine, and Cit, and their content in different alfalfa germplasm tissues ranged from 0.036 mg/g to 0.401 mg/g, accounting for approximately 42.0% of total amino acids. The OAAs content was the highest in flower tissue (**Figure 3F**; mean, 0.318 mg/g), followed by leaf tissue (mean, 0.107 mg/g), and the lowest in stem tissue (mean, 0.092 mg/g). There was a significant difference in OAAs among different germplasms and tissues, with a difference of approximately 10-fold; the content in 260dy flower tissue was the highest (0.401 mg/g), followed by that in 277bc flower tissue (0.390 mg/g) and 207kc flower tissue (0.374 mg/g). The OAA content in more than half of the alfalfa germplasms was less than 0.100 mg/g, and that in 22ywx stem tissue was the lowest (0.038 mg/g).

By comparing NVMs with different taste characteristics in different categories, the content distribution among the different germplasms was found to be significantly different. Overall, the stem tissue of alfalfa germplasm was rich in catechins and phenolic acids; the flower tissue was rich in flavonoids, SAAs, BAAs, and OAAs; and the leaf tissue was rich in UAAs and (UAAs+SAAs)/BAAs. Thus, the stem and leaf tissues could be used as raw materials to develop herbal tea to ensure the taste concentration and strength of the drink, and to promote freshness and sweetness. The stem tissue of germplasm 35zg was rich in catechins, the flower tissue of germplasms 207kc and 35zg was rich in flavonoids and phenolic acids, the flower tissue of 260dy was rich in SAAs and OAAs, the leaf tissue of 207kc was rich in UAAs, and the flower tissues of 277bc and 207kc were rich in BAAs.

### 3.2 Screening of differential NVMs among alfalfa (*Medicago sativa* L.) germplasms and tissues

#### 3.2.1 PLS-DA analysis

**Figure 4:**
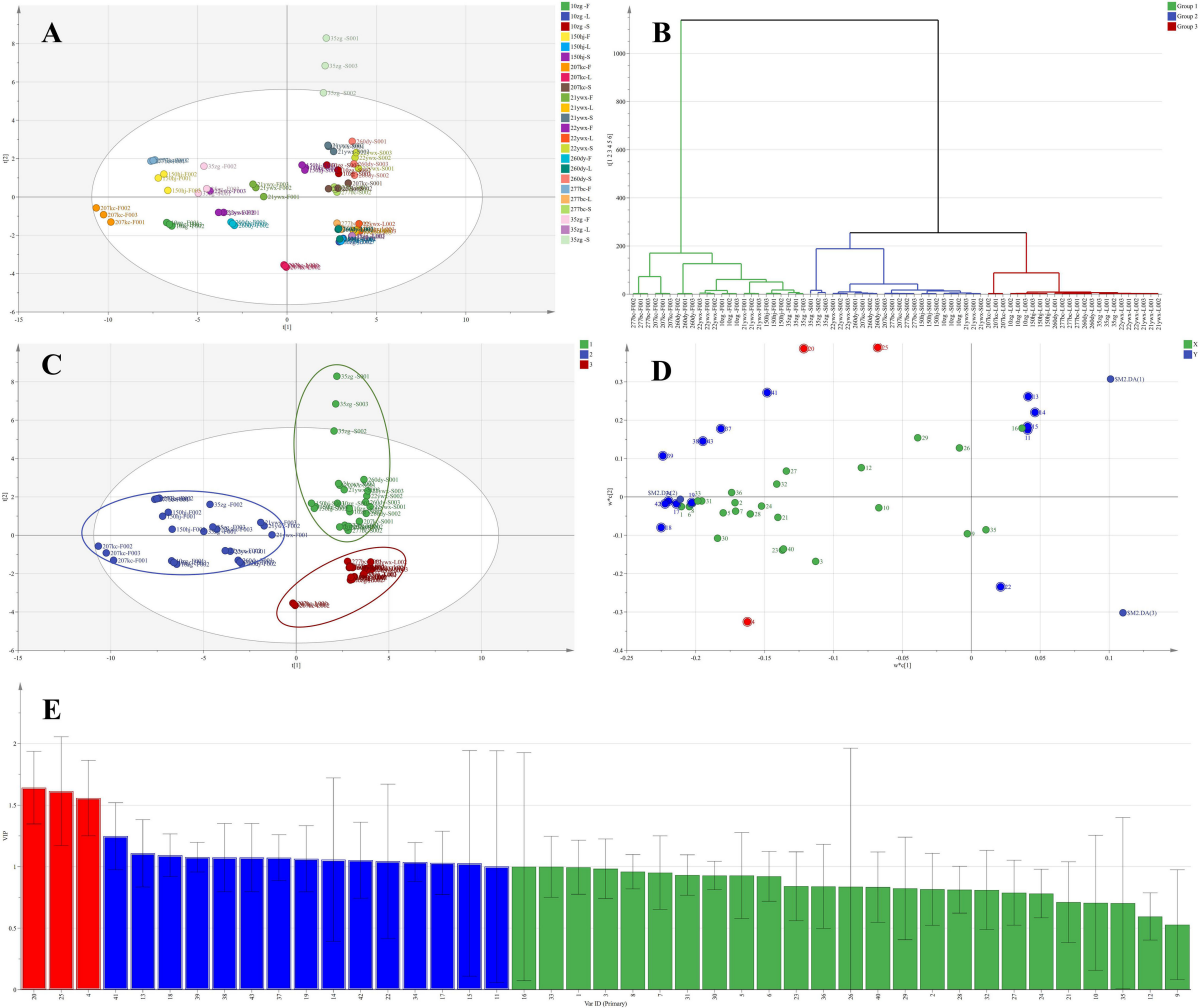
Analysis of differential non-volatile metabolites (NVMs) among alfalfa (*Medicago sativa L.*) germplasms and tissues. **(A)** partial least-squares discriminant analysis score scatter plots, **(B)** dendrogram, **(C)** cluster score scatter plots, **(D)** loading scatter plots, **(E)** variables important in the projection (VIP) plots.

PLS-DA is a statistical analysis method that simplifies multiple indicators into a small number of comprehensive indicators, using a few variables to reflect as much information as possible about the original variables, ensuring that the loss of original information is minimal and the number of variables is as small as possible. It is widely used in mining plant characteristic flavor compounds and screening of key metabolites with quality differences in different processed products (Hua et al., 2022; Wang et al., 2022b, 2023). To investigate the differences in NVMs among different alfalfa germplasms and tissues, PLS-DA analysis was conducted on 43 NVMs detected by targeted quantitative detection of flower, stem, and leaf tissues from eight alfalfa germplasms. The scatter plot (**Figure 4A**) showed significant differences in distribution among the different alfalfa samples, with greater differences among tissues than among germplasms. The stem tissue of the 35zg germplasm was located at the highest point on the upper right side, corresponding to its high content of catechins (**Figures 2A–C**), the leaf tissue of the 207kc germplasm was distributed at the lowest side, corresponding to its high SAAs and UAAs content (**Figures 2A–C**), and the flower tissue of the 207kc germplasm was distributed on the leftmost side, corresponding to its high content of flavonoids, phenolic acids, and BAAs (**Figures 2D, E**, **3D**). The R^2^X, R^2^Y, and Q^2^ values were 0.83, 0.92, and 0.88, respectively, indicating that the model was highly fitted and had a strong predictive ability.

Dendrogram clustering analysis (**Figure 4B**) showed that 24 alfalfa samples were clustered into three categories at a distance of 200 Euclidean: flower tissue category, leaf tissue category, and stem tissue category, whereas at a distance of 250 Euclidean, 24 alfalfa samples were clustered into two categories: flower tissue and leaf stem tissue. The difference in NVMs among alfalfa tissues was higher than that among germplasm, and the difference in flower tissues among the three tissues was significantly greater than that between the stem and leaf tissues. The differences between the tissue parts were further verified using a clustering score map (**Figure 4C**), where flower tissues were distributed on the left side, stem tissues were distributed on the upper right side, and leaf tissues were distributed on the lower right side.

The scatter plot (**Figure 4D**) clearly showed the differential distribution of NVMs in the alfalfa samples. The closer the distance to the sample point, the higher the NVMs content in that sample (Li et al., 2021; Wang et al., 2020). For example, the stem tissues were rich in EGCG, C, EC, EGC, and ECG; the leaf tissues were rich in isoflavones, L-Glu, and Ala; and the flower tissues were rich in quercetin, L-tryptophan, myricetin, quercetin 3-O-D-glucose-7-O-D-gentiobioside, Leu, Ile, His, and Cit.

#### 3.2.2 Heatmap analysis

VIP analysis was conducted to screen for key differential NVMs that could characterize the differences in the flower, stem, and leaf tissues of the eight alfalfa germplasms. Based on the principle of VIP > 1.0 (Hua et al., 2024b; Wang et al., 2022), 18 differential NVMs were screened (**Figure 4E**), including 10 amino acids (phenylalanine, L-tyrosine, Met, L-tryptophan, Ile, Leu, Cit, Val, His, and L-Glu), 4 catechins (EGCG, C, EC, and EGC), and 4 flavonoids (myricetin, prunin, quercetin 3-O-D-glucose-7-O-D-gentiobioside, and quercetin), which contribute to the growth of the eight alfalfa germplasm stems. Phenylalanine, L-tyrosine, and prunin had VIP values greater than 1.5, indicating that they were the most critical differential NVMs.

**Figure 5:**
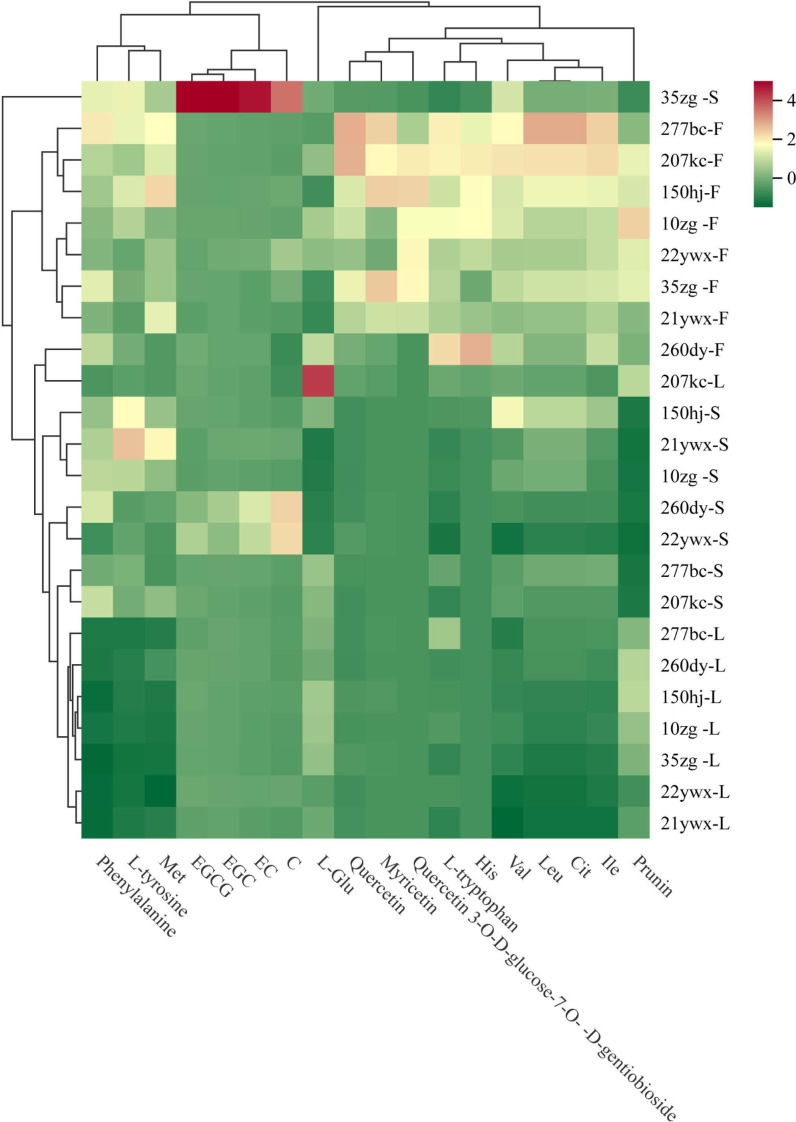
Heat map analysis of differential non-volatile metabolites (NVMs) among alfalfa (*Medicago sativa L.*) germplasms and tissues.

To further analyze the content distribution of differential NVMs in the 24 alfalfa samples, a heatmap (**Figure 5**) was created and clustered according to the alfalfa samples and differential NVMs. Alfalfa samples were divided into four categories: 35zg germplasm stem tissue clustered into one category, other stem tissues clustered together, flower tissues of different germplasms clustered together, and leaf tissues clustered together.

Differential NVMs were divided into five categories. The first category included three amino acid components (phenylalanine, bitter L-tyrosine, and sweet Met), which had the highest content in stem tissues of 35zg, 21ywx, and 150hj, as well as flower tissues of 277bc, 207kc, and 150hj, and the lowest content in leaf tissues of the eight alfalfa germplasms. The second category included all four catechins with strong taste intensity (EGCG, C, EC, and EGC), which had the highest content in 35zg stem tissue, followed by the stem tissue of 260dy and 22ywx, and there was no significant difference among the other alfalfa samples. The third category included one NVMs, umami L-Glu, which had the highest content in 207kc leaf tissue, the second highest content in 260dy flower tissue, and the lowest content in stem tissue of the eight alfalfa germplasms. The fourth category contained nine differential NVMs, including six amino acids (L-tryptophan, Ile, Leu, Val, His, and Cit with bitter taste) and three flavonoids (myricetin, quercetin 3-O-D-glucose-7-O-D-gentiobioside, and quercetin with astringent taste), with the highest content in the flower tissues of the 277bc, 207kc, and 150hj germplasms; the second highest content in the flower tissues of the 10zg, 22ywx, and 35zg germplasm, and the lowest content in the stem tissues of eight alfalfa germplasms. The fifth category included one NVMs (prunin), which had the highest content in 10zg flower tissue, the second highest content in the flower tissue of the 207kc, 150hj, 22ywx, and 35zg germplasm, the third highest content in the leaf tissue of the 277kc, 260dy, 150hj, and 10zg germplasm, and the lowest content in the stem tissue of the eight alfalfa germplasms.

### 3.3 Comparison analysis of differential NVMs among alfalfa (*Medicago sativa* L.) germplasms and tissues

#### 3.3.1 Comparison analysis of differential NVMs among tissues

**Figure 6:**
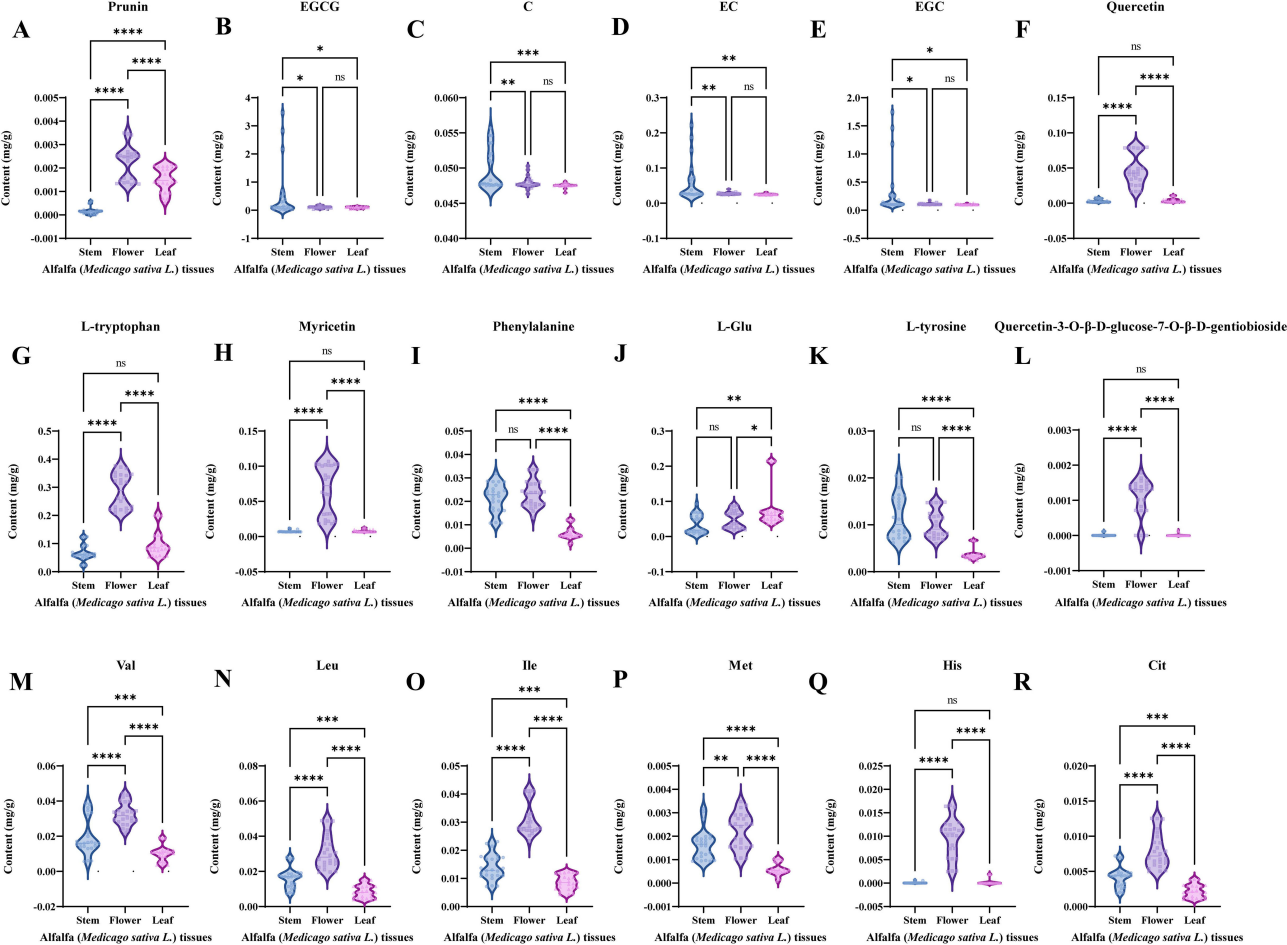
Violin diagram of key differential non-volatile metabolites (NVMs) among Alfalfa (*Medicago sativa L.*) tissues. **(A)** prunin content, **(B)** EGCG content, **(C)** C content, **(D)** EC content, **(E)** EGC content, **(F)** quercetin content, **(G)** L-tryptophan content, **(H)** myricetin content, **(I)** phenylalanine, **(J)** L-Glu content, **(K)** L-tyrosine content, **(L)** quercetin 3-O-D-glucose-7-O-D-gentiobioside content, **(M)** Val content, **(N)** Leu content, **(O)** Ile content, **(P)** Met content, **(Q)** His content, **(R)** Cit content.

To further analyze the differential distribution of differential NVMs in the tissues of the alfalfa germplasms, violin plots of 18 differential NVMs in different tissues were created by pooling data of one tissue type from eight germplasms (**Figure 6**).

A total of 18 differential NVMs showed significant differences distribution among three alfalfa tissue and could be divided into four categories: the first category included EGCG (**Figure 6B**), C (**Figure 6C**), EC (**Figure 6D**), and EGC (**Figure 6E**), and their contents were significantly highest in stem tissue, with no significant difference between leaf tissue and flower tissue; the second category included prunin (**Figure 6A**), Val (**Figure 6M**), Leu (**Figure 6N**), Ile (**Figure 6O**), Met (**Figure 6P**), and Cit (**Figure 6R**), and they showed significant differential distribution in all three tissue parts, with the highest content in flower tissue, followed by leaf tissue, and the lowest content in stem tissue; the third category included quercetin (**Figure 6F**), L-tryptophan (**Figure 6G**), myricetin (**Figure 6H**), quercetin 3-O-D-glucose-7-O-D-gentiobioside (**Figure 6L**), and His (**Figure 6Q**), and their contents were significantly highest in flower tissue, with no significant difference between leaf tissue and stem tissue; the fourth category included phenylalanine (**Figure 6I**) and L-tyrosine (**Figure 6K**), whose contents were significantly higher in stem and flower tissues than in leaf tissues, with no significant difference between stem tissue and flower tissue; the fifth category included L-Glu (**Figure 6J**), which had the highest content in leaf tissue and no significant difference between stem tissue and flower tissue.

Overall, flower tissues of alfalfa germplasm were rich in astringent NVMs, such as prunin, myricetin, and quercetin 3-O-D-glucose-7-O-D-gentiobioside, and bitter NVMs, such as Val, Leu, Ile, Met, Cit, and His (Hua et al., 2024a; Ouyang et al., 2024; Wang et al., 2021; Yu et al., 2023), meanwhile the content of umami NVMs, such as L-Glu, and taste intensity NVMs, such as EGCG, C, EC, and EGC, was relatively low, which hindered the development of new alfalfa flower tissue beverages. The stem and leaf tissues were rich in umami NVMs, such as L-Glu, as well as flavor-intensity NVMs, such as EGCG, C, EC, and EGC, whereas the content of bitter amino acids and bitter flavonoids was not high, which provided the freshness and strong taste of alfalfa herbal tea products (Wang et al., 2024; Yang et al., 2025).

#### 3.3.2 Comparison analysis of differential NVMs among *Medicago sativa* L. germplasms

**Figure 7:**
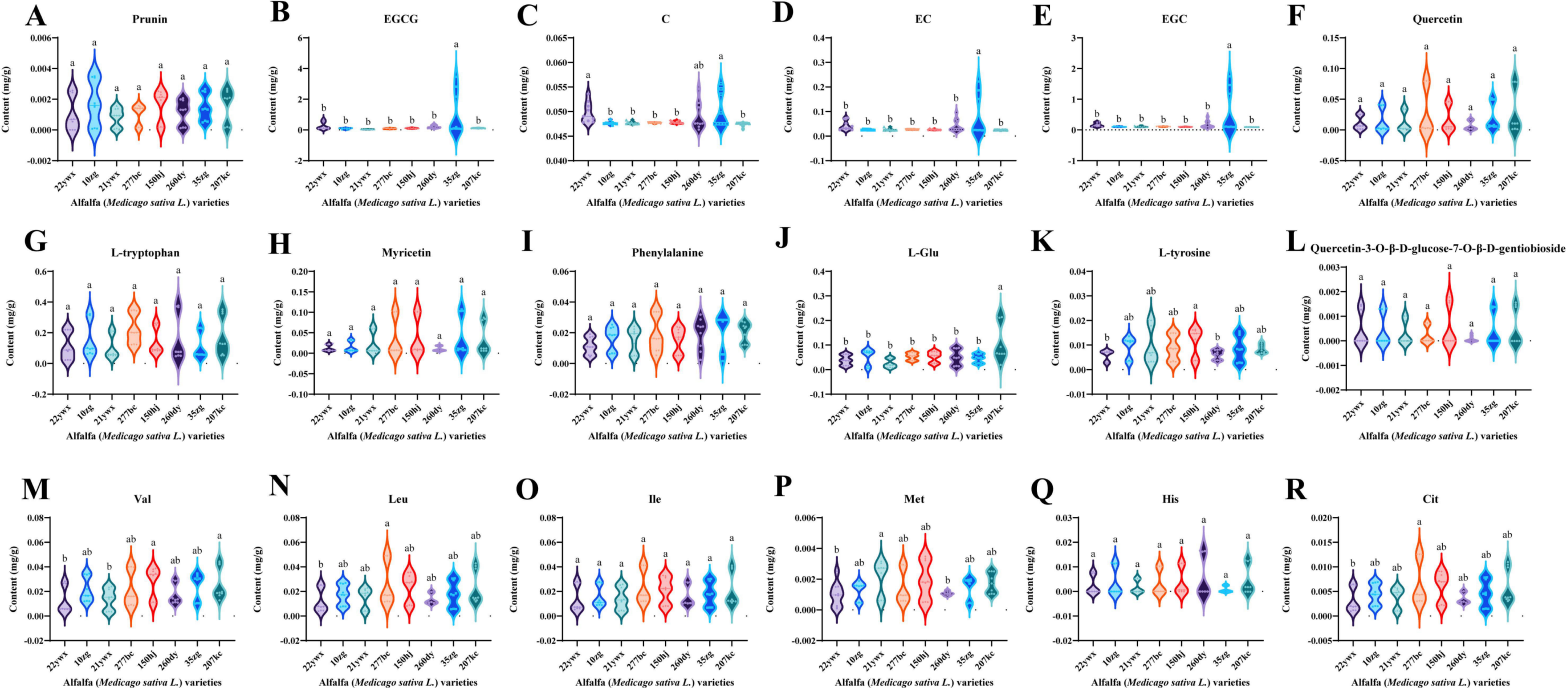
Violin diagram of key differential non-volatile metabolites (NVMs) among alfalfa (*Medicago sativa L.*) germplasms. **(A)** prunin content, **(B)** EGCG content, **(C)** C content, **(D)** EC content, **(E)** EGC content, **(F)** quercetin content, **(G)** L-tryptophan content, **(H)** myricetin content, **(I)** phenylalanine, **(J)** L-Glu content, **(K)** L-tyrosine content, **(L)** quercetin 3-O-D-glucose-7-O-D-gentiobioside content, **(M)** Val content, **(N)** Leu content, **(O)** Ile content, **(P)** Met content, **(Q)** His content, **(R)** Cit content.

To further analyze the differential distribution of differential NVMs in different alfalfa germplasms, violin plots of 18 different NVMs distributed in eight alfalfa germplasms were created by pooling data from three tissues of the same germplasm (**Figure 7**), and 18 differential NVMs were clustered into two categories.

The first category included eight NVMs: prunin (**Figure 7A**), quercetin (**Figure 7F**), L-tryptophan (**Figure 7G**), myricetin (**Figure 7H**), phenylalanine (**Figure 7I**), quercetin 3-O-D-glucose-7-O-D-gentiobioside (**Figure 7L**), Ile (**Figure 7O**), and His (**Figure 7Q**), and there were no significant differences among the eight alfalfa germplasms. The second category included 10 NVMs, such as EGCG (**Figure 7B**), C (**Figure 7C**), EC (**Figure 7D**), EGC (**Figure 7E**), L-Glu (**Figure 7J**), L-tyrosine (**Figure 7K**), Val (**Figure 7M**), Leu (**Figure 7N**), Met (**Figure 7P**), and Cit (**Figure 7R**), and they showed significant differential distribution in 8 alfalfa germplasms, with no significant differences in flavonoids among different alfalfa germplasms, but significant differences in catechins and amino acids. The 10 key differential NVMs were divided into three categories: the first category included catechins, such as EGCG, C, EC, and EGC, which had the highest content in 35zg germplasm and no significant differences in other germplasms; the second category included L-Glu, which had the highest content significantly in the 207kc germplasm and no significant difference in other germplasms; the third category included L-tyrosine, Val, Leu, Met, and Cit, with the significantly lowest content in 22ywx germplasm, L-tyrosine and Val were significantly highest in 150hj germplasm, Leu and Cit were significantly highest in 277bc germplasm, and Met was significantly highest in 21ywx germplasm.

Overall, the 35zg germplasm was rich in taste intensity NVMs, such as EGCG, C, EC, and EGC, whereas the content of bitter NVMs, such as L-tyrosine, Val, Leu, and Cit, was low. The 207kc germplasm was rich in umami NVMs, such as L-Glu and Met, and the content of bitter NVMs was low. Both alfalfa germplasms are thus suitable for the future development of new alfalfa herbal tea products. Germplasms 150hj and 277bc had high levels of bitter NVMs, such as Leu, Cit, L-tyrosine, and Val, and low levels of flavor-positive NVMs, such as L-Glu with an umami taste and EGCG, C, EC, and EGC with an intense taste, which resulted in high bitterness and low freshness, making them unsuitable for the development of new alfalfa herbal teas.

## 4 Conclusions

Key quality NVMs showed significant differences in distribution among different alfalfa germplasms and tissues. A total of 43 NVMs were quantitatively detected using UHPLC-Q Exactive-MS. Six catechin components were identified, with EGCG content being the highest among these. Comparing different tissues, the stem tissue was rich in catechins and phenolic acids; the flower tissue was rich in flavonoids, SAAs, BAAs, and OAAs; and the leaf tissue was rich in UAAs and (UAAs+SAAs)/BAAs. Comparing different germplasms revealed that 35zg germplasm stem was rich in catechins, 207kc flower was rich in flavonoids, phenolic acids, and BAAs; 260dy flower was rich in SAAs and OAAs; and 207kc leaf was rich in UAAs. Through significant difference analysis, VIP analysis, and heat map analysis, 18 differential NVMs—key metabolites causing differences in alfalfa germplasm and tissues—were screened. Tissue classification analysis revealed that alfalfa flower tissues were rich in astringent NVMs, such as prunin, myricetin, and quercetin 3-O-D-glucose-7-O-D-gentiobioside, as well as bitter NVMs including Val, Leu, Ile, Met, Cit, and His; stem and leaf tissues were rich in umami NVMs (such as L-Glu) and flavor-intensity NVMs (such as EGCG, C, EC, and EGC). Germplasm classification analysis revealed 10 key differential NVMs; 35zg germplasm was rich in NVMs (EGCG, C, EC, and EGC), whereas the content of L-tyrosine, Val, Leu, and Cit with bitter taste was low; 207kc germplasm was rich in L-Glu and Met with umami taste, and the content of bitter NVMs was low; thus, both germplasms were suitable alfalfa materials for developing new herbal tea products. This study provides theoretical and technical support for analyzing the intrinsic characteristics and diversified utilization of alfalfa materials, while facilitating the development of characteristic herbal tea products. Subsequent research could deepen the detection of volatile metabolites, optimize processing technologies, and develop high-quality characteristic products of alfalfa herbal tea to improve and efficiently utilize alfalfa materials.

## Data Availability

The original contributions presented in the study are included in the article/**Supplementary Material**. Further inquiries can be directed to the corresponding authors.
